# The Key Roles of Interferon Lambda in Human Molecular Defense against Respiratory Viral Infections

**DOI:** 10.3390/pathogens9120989

**Published:** 2020-11-26

**Authors:** Alexey A. Lozhkov, Sergey A. Klotchenko, Edward S. Ramsay, Herman D. Moshkoff, Dmitry A. Moshkoff, Andrey V. Vasin, Maria S. Salvato

**Affiliations:** 1 Peter the Great St. Petersburg Polytechnic University, 195251 St. Petersburg, Russia; aswert6@mail.ru (A.A.L.); moshkoffd@gmail.com (D.A.M.); andrey.vasin@influenza.spb.ru (A.V.V.); 2 Smorodintsev Research Institute of Influenza, Russian Ministry of Health, 196376 St. Petersburg, Russia; fosfatik@mail.ru (S.A.K.); influenza.spb@gmail.com (E.S.R.); 3 Russian Technological University (MIREA), 119454 Moscow, Russia; dMoshkoff@uspharmabio.com; 4 US Pharma Biotechnology, Inc., 5000 Thayer Center, Suite C, Oakland, MD 21550, USA; 5 Global Virus Network(GVN), 725 W Lombard St, Baltimore, MD 21201, USA; 6 St. Petersburg State Chemical-Pharmaceutical Academy, 197022 St. Petersburg, Russia; 7 Institute of Human Virology, University of Maryland School of Medicine, Baltimore, MD 21201, USA

**Keywords:** respiratory viruses, influenza, innate immunity, interferons-α/β, interferons-λ, interferon stimulated genes

## Abstract

Interferons (IFN) are crucial for the innate immune response. Slightly more than two decades ago, a new type of IFN was discovered: the lambda IFN (type III IFN). Like other IFN, the type III IFN display antiviral activity against a wide variety of infections, they induce expression of antiviral, interferon-stimulated genes (*MX1*, *OAS*, *IFITM1*), and they have immuno-modulatory activities that shape adaptive immune responses. Unlike other IFN, the type III IFN signal through distinct receptors is limited to a few cell types, primarily mucosal epithelial cells. As a consequence of their greater and more durable production in nasal and respiratory tissues, they can determine the outcome of respiratory infections. This review is focused on the role of IFN-λ in the pathogenesis of respiratory viral infections, with influenza as a prime example. The influenza virus is a major public health problem, causing up to half a million lethal infections annually. Moreover, the virus has been the cause of four pandemics over the last century. Although IFN-λ are increasingly being tested in antiviral therapy, they can have a negative influence on epithelial tissue recovery and increase the risk of secondary bacterial infections. Therefore, IFN-λ expression deserves increased scrutiny as a key factor in the host immune response to infection.

## 1. Introduction

### 1.1. Virus Entry Triggers Host Signaling Responses

**Figure 1:**
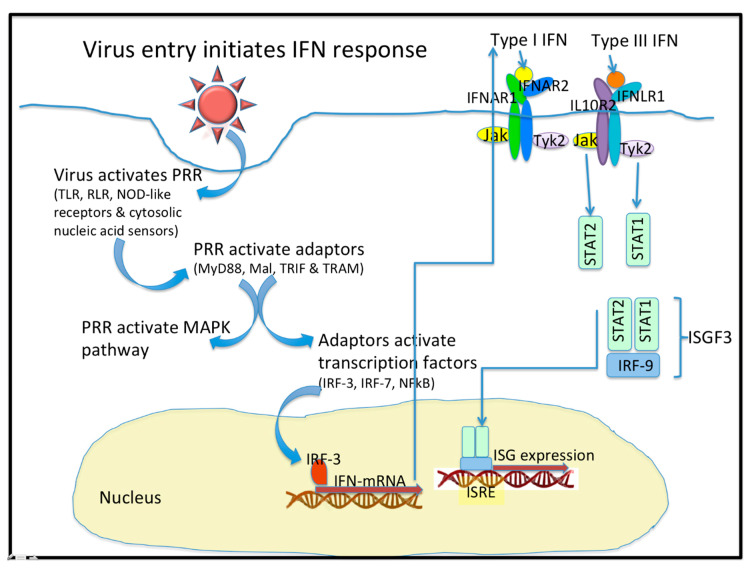
Virus entry initiates the interferon (IFN) response. Upon entering a cell, virus activates Pattern Recognition Receptors (PRR) that include the Toll-like receptors (TLR), the RIG-I-like receptors (RLR), the NOD-like receptors, and some cytosolic nucleic acid receptors. These PRR activate adaptor molecules like MyD88, Mal, TRIF, and TRAM. PRR also activate the MAPK pathway. The Adaptor molecules activate Transcription factors like IRF-3, IRF-7 and NF-kB. IFN mRNA are transcribed and they express the type I, type II (not shown), and type III IFN. Type I and Type III IFN bind to distinct receptors that activate similar signaling pathways and transcriptional responses. The heterodimeric IFN receptors signal through the JAK/STAT pathways to form a complex with IRF-9 to initiate the expression of hundreds of Interferon stimulated genes (ISG). The ISG are peptidic antivirals that interfere with virus replication.

In viral infection, the protective barriers are host skin and mucous membranes. In the initial stages of a viral infection, quick activation of a non-specific immune response occurs in response to the infiltration in a cell (Figure 1). Cells utilize various pattern recognition receptors (PRR) to detect viral particles. Toll-like receptors (TLR) are one such sensor. RIG-I-like receptors (RLR), Nod-like receptors, and cytosolic nucleic acid sensors become involved when viral particles enter the cytoplasm [1]. To date, ten TLR types have been found in humans. It is well known that TLR-3, TLR-7, TLR-8, and TLR-9 are localized in endosomes, while others reside on the outer surface of the cytoplasmic membrane [1,2]. Signaling through TLR requires the use of various combinations of the following protein adapters: MyD88, Mal, TRIF, and TRAM [2]. Activation of molecular adapters is aimed at regulating the activity of the NFκB, IRF-3, and IRF-7 transcription factors, and activation of MAPK-dependent signaling pathways. The combined action of these transcription factors with the AP-1 protein effectively induces the expression of target genes [1,3].

Cells are capable of TLR-independent responses to pathogen infiltration, and such responses are mediated through cytosolic sensors. The most important sensors are RNA helicases that belong to the RLR family: RIG-I, MDA-5 and LGP-2 [1]. The activated multimeric forms of RIG-I or MDA-5 are able to interact with the MAVS protein adapter located on the outer mitochondrial membrane or in peroxisomes [1]. Viral dsRNA activates both RIG-I and MDA-5. RNA containing a 5′-triphosphate end, without a cap structure, can also activate RIG-I. The MAVS protein adapter plays the role of a scaffold protein and is involved in the recruitment of signaling cascade components aimed at activating both NFκB and IRF-3 [3].

### 1.2. IFN Are Class II Cytokines

Class II cytokines are an extensive family of protein mediators that have similar gene structure, receptor structure and common signaling pathways. Four types of cytokines are commonly assigned to this family: “IL-10-like” cytokines; and the type I, II, and III interferons (IFN) [4]. Class II cytokine receptors are heterodimers and consist of a subunit featuring high ligand affinity (usually referred to as R1) and a low-affinity subunit (R2). Both subunits, however, are necessary for signal transmission [4]. IFN play a crucial role in the immune response. They inhibit the spread of viral infection in the early stages of illness and form the first line of defense in mammals against viral infections [5,6]. All IFN have an α-helical structure. According to the amino acid sequence and the type of receptor through which signal transmission is mediated, IFN are divided into three groups [3,6,7].

The most studied are type I IFN. In humans, a number of genes have been identified: 13 genes encoding different IFN-α subtypes; 1 gene encoding IFN-β; and other genes encoding IFN-ω, IFN-τ, IFN-ε, IFN-δ and IFN-κ [8]. Despite the wide variety of type I IFN, their action is mediated through the ubiquitous, heterodimeric IFNα receptor (IFNαR); such action is aimed at the induction of interferon-stimulated genes (ISG) [6]. The biological activity of type I IFN depends on their affinity for receptor subunits and the density of these subunits on the cell surface [9]. For example, all IFN-α subtypes are characterized by a non-optimal affinity for the subunit of the receptor [4], which leads to differences in biological activity between α and β IFN [10]. Therefore, the effects of type I IFN may vary in duration and intensity [9].

IFN induce activation of defense mechanisms and prepare cells for possible viral invasion. Induction of IFN production is closely associated with PRR activation. Generally, a cell first synthesizes IFN-β in response to signs of infection. Activation of the transcription factors NFκB and IRF-3 is required to this end. IFN-β stimulates the production of other IFN through its autocrine action associated with activation of IRF-7. IRF-7, in turn, binds to IFN-β and IFN-α gene promoters, enhancing the synthesis of those cytokines [3,11].

Type I IFN interact with the heterodimeric IFNα receptors. Ligand binding causes dimerization of receptor subunits and activation of tyrosine kinases JAK1 and Tyk2, which phosphorylate the transcription factors STAT1 and STAT2 [3,12]. Due to interaction with IRF-9, the ISGF3 heterotrimeric complex is formed. The complex is bound by the ISRE regulatory element that is located on the promoters of most ISG. Consequently, type I IFN enhance the transcription of hundreds of genes and contribute to the cell’s antiviral response [3,13]. It is important to note that the expression of an entire ensemble of genes is necessary to limit viral replication; expression of single genes alone is not capable of providing a sufficient antiviral response [3]. The signaling pathways that are affected by the actions of type III IFN are generally similar to those of type I IFN [14].

It has been shown that other signaling pathways can affect the induction of IFN-dependent transcription. The JAK-STAT signaling pathway is known to be associated with the phosphotidylinositol-3-dependent signaling cascade [12,15]. Moreover, the effects of type I IFN are associated with increased activity of MAPK-dependent signaling cascades [3,16]. In addition to direct antiviral effects, type I IFN have immuno-modulatory properties [3,9,17,18,19].

Cells have mechanisms for inhibiting the effects of type I IFN. These mechanisms may be focused on the attenuation of JAK-STAT-dependent signaling cascades. For example, it has been shown in vivo that injections of IFN-α cause activation of negative regulators, such as SOCS-1 and SOCS-3 [20,21]. Moreover, a prolonged effect of the USP18/UBP43 inhibitor ISG-encoded isopeptidase has been described [20]. The USP18/UBP43 protein is an ISG15-specific protease [22]. Knockout of USP18/UBP43 in mice results in hypersensitivity to type I IFN. This protein inhibits JAK-STAT signaling pathways by binding to the IFNαR2 subunit and blocking the interaction between the JAK1 kinase and the heterodimeric receptor [23]. Pre-incubation with type I or type III IFN has been shown to cause desensitization of cells to the stimulatory effects of IFN-α. The degree of desensitization depends on the expression level of USP18/UBP43. Thus, there is a negative feedback loop attenuating both the type I and type III IFN [9].

## 2. IFN-λ Play a Distinct Anti-Viral Role in Collaboration with Other IFN

### 2.1. IFN-λ Structure

Type III IFN (IFN-λ) are a group of cytokines that is related to type I IFN and elicit similar antiviral effects [1,24]. Four IFN-λ subtypes have been found in humans: IFN-λ1 (IL-29), IFN-λ2 (IL-28A), IFN-λ3 (IL-28B), and IFN-λ4. All of these proteins are encoded on the 19th chromosome, and these genes consist of five or six exons [7]. Several of these IFN (λ1, λ2, λ3) feature a high degree of amino acid conservation [25,26], which suggests the presence of a single ancestor [7,27]. IFN-λ2 and IFN-λ3, for example, are 96% identical in amino acid sequence; they differ by just seven residues. However, IFN-λ1 is only 80% identical to them in primary structure and also differs in disulfide bond configuration; IFN-λ1 does not form a third disulfide bond [4,25]. Initially, IFN-λ4 was thought to be a pseudogene. However, it is now known that humans have the *IFNL4* gene, but in some populations, there is a polymorphism (ss469415590, TT/ΔG). The TT allele causes a frameshift leading to suppression of *IFNL4* production, while the ΔG allele results in the functional *IFNL4* gene [4,28]. Although the product of this gene is only 40.8% identical to IFN-λ3 [29], IFN-λ4 interacts with the heterodimeric receptor common to all IFN-λ. It has been revealed that N-glycosylation of IFN-λ4 is necessary for protein secretion. IFN-λ1 is also known to have a potential N-glycosylation site at asparagine residue 65 [4]. Only IFN-λ2 and IFN-λ3 have been found in mice, and murine IFN-λ1 is a pseudogene [26,30,31]. Murine IFN-λ2 and IFN-λ3 are also N-glycosylated [27]. IFN-λ are conserved in tetrapod vertebrates; and tetrapod type III IFN form a monophyletic group [32]. IFN-λ are considered to be derived from “IL-10-like” cytokine family [33]. Fundamental similarities between type III IFN systems of mammals and birds indicate that type III IFN might play a significant role in defending mucosal surfaces against viral infections in birds [34]. Two groups of IFN were discovered in fishes, but fish IFN are evolutionarily closer to type I than to type III tetrapod IFN [33].

Although type III IFN (IFN-λ) should be considered most closely related to type I IFN (according to primary structure), IFN-λ are close to IL-10 (and other “IL-10-like” cytokine family members) in their spatial structure [25,26]. The spatial structure of IFN-λ includes five α-helices (A, C, D, E, F) and an element B having a less-defined structure [4]. Helix A, helix F, and the AB loop are responsible for the interaction between lambda IFN and their receptors. Certain amino acids located in the AB loop (Lys49 and Arg51 for IFN-λ3; Arg49 and His51 for IFN-λ2) have a critical effect on affinity for the IFNλR1 subunit of the receptor. Helix D is responsible for binding to the IL-10R2 subunit (Gly95 for IFN-λ3, and Val95 for IFN-λ2, are considered to be important for the interaction). Lambda IFN differ in their receptor subunit affinities; the stabilities of their final ligand-receptor complexes also differ [29]. Hepatitis C virus (HCV) persistence is strongly associated with the expression of a functional *IFNL4* gene, whereas the nonfunctional *IFNL4* gene is associated with more rapid viral clearance [28]. Humans have several mechanisms to limit the expression of functional IFN-λ4 through noncoding splice variants and nonfunctional protein isoforms. Moreover, protein-coding IFN-λ4 mRNA are not loaded onto polyribosomes and lack a strong polyadenylation signal, which results in poor translation efficiency [35]. Amino-acid substitution (P70S) is also strongly associated with HCV clearance. Patients harbouring the S70 variant display lower ISG expression, better treatment response rates and better spontaneous clearance rates, compared with patients coding for the fully active P70 variant [36]. Interestingly, variant E159 (E159K substitution) of IFN-λ4, that was found in some ancient African populations, exhibit more significant antiviral activity than wild-type IFN-λ4. Thus, substitution E154K also negatively affects IFNλ4 activity by reducing its secretion and potency [37].

### 2.2. Expression of IFN-λ

Synthesis of type III IFN is induced by viral infection and PRR activation (TLR, RLR, Ku70), and it occurs in various tissues. For example, high IFN-λ levels are observed in the lungs and liver [25,26,38]. Many cell types are capable of producing both IFN-α and IFN-β (IFN-α/β) with IFN-λ, but there are exceptions. For example, in response to infection with influenza virus or herpes simplex virus type-2 (HSV-2), macrophages can produce only IFN-α/β, but not IFN-λ [30,39]. Infection with swine influenza virus (H3N2) lead to IFN-β, but neither IFN-α nor IFN-λ1 expression in porcine macrophages [40]. IFN-λ expression has been discovered in respiratory epithelial cells, keratinocytes, dendritic cells, hepatocytes, and primary neuronal cells [30]. Moreover, IFN-λ are the most common IFN produced by respiratory epithelium in response to dsRNA (poly(I:C), a TLR-3 agonist); agonists of other TLR do not induce production of IFN-α/β or IFN-λ in this cell type [38]. A potent IFN-λ response is observed upon infection of human respiratory epithelial cells with respiratory viruses, such as influenza or rhinovirus [30,38,39,41,42]. Additionally, swine influenza virus (H3N2) up-regulates IFN-λ1 in porcine epithelial cells as well as in precision-cut lung slices [40]. However, myeloid dendritic cells (mDC) and plasmacytoid dendritic cells (pDC) appear to be the major producers of IFN-λ [43]. It was shown that CpG DNA (a TLR-9 agonist) induces the expression of IFN-α/β and IFN-λ in pDC, while lipopolysaccharides (LPS) and poly(I:C) (TLR-4 and TLR-3 agonists, respectively) induce expression of IFN-β and IFN-λ1-3 in mDC (but not IFN-α) [29].

### 2.3. Molecular Mechanism of IFN-λ Induction

IFN-λ are induced by pathways and factors similar to those involved in the induction of IFN-α/β. Moreover, IFN-λ production is mediated by activation of the same PRRs as IFN-α/β [26]. For example, expression of IFN-λ is significantly mediated by activation of the RIG-I and MDA-5-dependent signaling pathways in respiratory and dendritic cells [5,44,45]. However, it was found that the production of IFN-λ1, but not type I IFN, can also be induced by the activation of certain DNA sensors (Ku70). Induction of IFN-λ1 synthesis, in this case, is mainly associated with activation of the IRF-1 and IRF-7 transcription factors [46].

It was shown that the transcription factors IRF-1, IRF-3, IRF-7, and NFκB can bind to the promoters of *IFNL* genes. The synergistic effect of these transcription factors allows for maximum induction efficiency [45]. However, it is worth noting that expression of IFN-λ2 and IFN-λ3, like IFN-α, is still predominantly regulated by IRF-7 and NFκB; expression of IFN-λ1, like IFN-β, requires the combined action of IRF-3, IRF-7, and NFκB [47]. In mice lacking IFN-λ1, IRF-3 is not involved in the induction of IFN-λ expression in response to metapneumovirus infection [5,44]. Additionally, the Med23 subunit of the eukaryotic multiprotein mediator, which interacts with transcription factors and RNA polymerase II, binds directly to IRF-7 and induces IFN-λ synthesis. However, Med23 is not able to enhance the IRF-7-mediated induction of IFN-β transcription. These data emphasize an additional selectivity of the IFN response [48]. A detailed review of type I and type III IFN induction mechanisms, and their differences, has been published [26].

Expression kinetics for IFN-λ depend on cell type and induction conditions. In PBMC and fibroblasts, it has been shown that peak IFN-λ3 expression occurs 24 h after infection with cytomegalovirus, while the IFN-λ1 peak is 6 h after infection [49]. When primary human hepatocytes are infected with HCV, increased IFN-λ4 mRNA levels can be detected 2–4h after infection. However, the expression subsides after 8 h, which may suggest either: absence of a positive-regulation feedback loop; or (conversely) induction of specific negative-feedback mechanisms [29]. There is limited information about IFN-λ negative regulation (reviewed in [50]). Stimulation by type III IFN leads to ISG expression that includes *SOCS* and *IL-10* expression. Excessive SOCS-1 expression is associated with reduced STAT1 phosphorylation as well as reduced ISG expression [51]. Type III IFN activity may be inhibited in the presence of IL-10 [52]. Additionally, it should be noted that the level of IL10R2 subunit is modulated by ubiquitination leading to degradation of nonspecific subunits [53].

### 2.4. The IFN-λ Receptor (IFNλR)

IFN-λ actions are carried out through the heterodimeric IFNλR, consisting of the IFNλR1 and IL10R2 subunits [25,54]. The IL10R2 subunit is also part of the receptor complexes for IL-10, IL-22, and IL-26; it is expressed in cells of various tissues. Interestingly, the IFN-λ1 and IL-10-like cytokines bind with low affinity to the IL10R2 subunit itself. In turn, IFN-λ1 specifically binds to the IFNλR1 subunit in a 1:1 stoichiometric ratio [25]. An IFNλR has also been found in mice; the murine IFNλR amino acid sequence is approximately 67% similar to the human. It should be noted that both murine and human IFN-λ act non-specifically in terms of host species: murine IFN-λ can bind to human IFNλR [27]. On the other hand, murine and human IFN-λ exhibit some species specificity. For instance, murine IFN-λ3 is 51 times less active in human A549 cells than in mouse LKR10 cells. However, IFN-λ4 is more active in mouse cells [55].

Expression of the IFNλR1 subunit demonstrates restricted cellular distribution. For example, IFN-λ does not act on fibroblasts, splenocytes, macrophages, or (migrated, bone marrow-originating) endothelial cells, since IFNλR1 is not expressed in these cells, while IFN-α is able to activate all of them [27,30]. High IFNλR1 expression has been found in the lungs, intestines, liver, and upper epidermis [30]. Expression of IFNλR1 is mainly restricted to: epithelial cells [11], keratinocytes [56], differentiated dendritic cells (pDC and mDC) [57,58], and hepatocytes [59]. In monocytes and B cells, low levels of IFNλR1 expression are detected. Thus, these cells respond extremely weakly to IFN-λ [60]. As such, mucous membranes of the respiratory and gastrointestinal tracts are the primary tissue targets of IFN-λ [11]. This tissue specificity correlates with IFN-λ antiviral activity, which is seen mainly with viruses featuring high tropism for epithelial cells, like Orthomyxoviridae, Pneumoviridae, Coronaviridae, Picornaviridae, Herpesviridae, Flaviviridae, Reoviridae, Arenaviridae, Caliciviridae (Table 1. Viruses affected by IFN-lambda (Type III interferons)) [30]. In general, type III IFN control infection at mucosal barrier sites, while type I IFN are important for broad systemic infection control.

### 2.5. The Effects of IFN-λ on Cells

Lambda IFN are secreted into the extracellular space and exert autocrine or paracrine effects by binding to cell surface receptors. Although IFN-α/β and IFN-λ actions are realized through different receptors, they lead to the activation of similar signaling pathways. Upon IFN-λ binding, receptor subunits dimerize leading to activation of JAK/STAT-dependent signaling pathways: activation of JAK1 and Tyk2 tyrosine kinases; phosphorylation of receptor subunits; recruitment and subsequent phosphorylation of STAT1 and STAT2 proteins, and to a lesser extent STAT3-STAT5; and formation of the ISGF3 transcription complex. Interestingly, STAT1 can also be activated by the actions of various cytokines, while STAT2 phosphorylation is caused by the specific action of type I or type III IFN [72]. The ISGF3 complex is also formed in response to the actions of type I IFN. Therefore, IFN-λ functions significantly overlap with type I IFN functions and cause the expression of similar ISG. In addition to activating JAK/STAT signaling cascades, IFN-λ also influences MAPK signaling pathways, including the Erk, Jnk, and p38 kinases [14,26,73,74].

There are some differences in the mechanisms activated by type I and type III IFN. In intact cells, IFN-λ activate STAT-dependent signaling pathways slightly more weakly than IFN-α, which is associated with higher basal type I IFN levels. Interestingly, the JAK2 kinase (necessary for phosphorylation of STAT1) is specifically activated in response to IFN-λ; this may underlie the differences in IFN-λ and IFN-α/β effects. Moreover, gene knockout of JAK2, or its inhibition by substances (such as AG490 or 1,2,3,4,5,6-hexabromocyclohexane), can specifically block IFN-λ-dependent signaling cascades without affecting type I IFN-depended signaling pathways [75].

### 2.6. Immuno-Modulatory Activity of IFN-λ

IFN-λ-mediated signaling also regulates the immune response. The presence of phosphorylated STAT-3, STAT-4, and STAT-5 forms indicates the existence of an additional level of complexity. Thus, there are several associations: STAT3 phosphorylation is also a signaling mechanism used by members of IL-10-like cytokines (IL-10, IL-19, IL-20); phosphorylated forms of STAT5 are often associated with IL-2, IL-3, and GM-CSF; and STAT4 is associated with a T helper type 1 (Th1)-mediated immune response. In general, an entire body of knowledge indicates the presence of additional immuno-modulatory activities of IFN-λ [52].

In early studies, it was shown that IFN-λ1 causes the secretion of IL-6, IL-8, and IL-10 in PBMC. Selective blocking of IL-10 with specific antibodies leads to a decrease in the required dose of IFN-λ1 for the secretion of IL-6. In turn, the addition of IL-10 reduced the effects of IFN-λ1. Therefore, the existence of a feedback mechanism can be assumed by which IFN-λ1 causes the secretion of IL-10, which inhibits the effects of the former. This mechanism may be associated with competition between IFN-λ1 and IL-10 for binding to the IL10R2 subunit [76]. The IL-22 receptor also contains the IL10R2 subunit. IL-22 acts synergistically with IFN-λ and causes activation of STAT1-dependent signaling pathways in the suppression of rotavirus infections [77]. Dendritic cells express IFNλR during differentiation from monocytes. The dendritic cells, maturing upon stimulation with IFN-λ, induce IL-2-dependent proliferation of a population of CD4+/CD25+/Foxp3+ regulatory T cells [74].

## 3. Antiviral Effects

### 3.1. IFN-λ Are Universal Antivirals

IFN-λ play an important role in viral replication control (Table 1. Viruses affected by Type III Interferons). Although type III IFN are well known for their antiviral effects, IFN-λ also take part in the immune response to bacterial pathogens [78,79]. The transcriptional response to lambda IFN is generally weaker than that from type I IFN, but it is characterized by a longer duration [26]. The ability of lambda IFN to activate a narrower set of genes, in a restricted group of target cells expressing IFNλR, makes this IFN type a promising therapeutic agent [30]. The effects of IFN-λ are mainly directed at viruses thattarget cells of the respiratory system, gastrointestinal tract, urogenital tract, and liver [26]. It should be mentioned that lambda IFN display antiviral activity against a wide variety of viruses. For instance, IFN-λ3 is a key regulator of ISG expression upon infection of PBMC and fibroblasts with human cytomegalovirus [49]. Induction of lambda IFN inhibits HSV-1 replication in human lung (A549) cells [48] and HSV-2 in human cervical endothelial (End1/E6E7) cells [80]. It has also been shown that lambda IFN exhibit antiviral effects in persistent norovirus infections [68]. Dengue virus infection induces the production of IFN-λ1 in dendritic cells and lung epithelial cells. Blocking IFN-λ1-mediated signaling reduced dendritic cell migration by inhibiting CCR-7 expression [66]. IFN-λ1 and IFN-λ2 inhibited viral replication in a dose-dependent manner and increased the levels of antiviral ISG (Mx1 and OAS) [67]. It also has been shown that IFN-λ2 and IFN-λ3 elicit an antiviral effect against lymphocytic choriomeningitis virus in A549 cells. For IFN-λ2, it was noted that antiviral effects are realized only in the early stages of infection. The virus is able to reduce the expression of the IFNλR1 subunit in infected cells and attenuate IFN-λ-mediated signaling cascades [64]. A number of other examples of IFN-λ antiviral activity have been reviewed elsewhere [7,26,30].

Infection with hepatitis B virus (HBV) or HCV is associated with increased production of lambda IFN [26]. Moreover, significant IL10R2 and IFNλR1 expression has been detected in primary hepatocytes [71] and in a number of cell lines (Huh7, HepG2, Hep3B) [81]. IFN-λ1 and IFN-α induce similar antiviral ISG [81]. However, these responses feature different kinetics. IFN-α cause a rapid peak in ISG that is followed by a similar sharp decline; lambda IFN are characterized by a prolonged action [7,82]. Serum IFN-λ1 levels exceeded IFN-λ2 and IFN-λ3 levels more than twofold [83] and IFN-λ1 significantly reduced viral load during infection with HBV or HCV [71,81]. In turn, IFN-λ2 has regulatory activities and is capable of down-regulating the expression of hundreds of genes associated with: mRNA transcription regulation, nucleic acid binding, and homeobox transcription factors [81]. It has been shown that in patients with allele (T/T) at the rs12979860 polymorphism (SNP near *IFNL3*), serum IFN-λ levels were lower and that this allele is associated with negative (treatment) outcome [65,83,84]. In contrast, spontaneous clearance of infection was associated with higher IFN-λ1 level and allele (C/C) at rs12979860 [83]. Moreover, chronic HCV patients are characterized by low IFN-λ1 levels. A possible reason may be inhibition of IFN-λ1 production due to the actions of viral proteins NS3 and E2 on dendritic cells [83]. Thus, it is possible that hepatitis viruses have mechanisms that attenuate IFN-λ’s protective effects.

Lambda IFN play a key role in protection against rotavirus, which has a pronounced tropism for intestinal epithelial tissues [85]. It has been shown in vivo that intestinal epithelial cells are capable of producing Mx1 in response to IFN-λ-mediated stimulation in larger amounts than under IFN-α/β stimulation. Subcutaneous administration of IFN-λ2 to mice reduced viral load in the intestinal epithelium in a dose-dependent manner [31]. Severe acute respiratory syndrome coronavirus (SARS-CoV) titers were increased in the lungs of double knockout mice (*IΦNAP1*, *IFNLR1* genes), and viral particles were found even in their litters. Collectively, these facts emphasize the non-redundancy of lambda IFN in the control of viral replication in epithelial cells of both the intestines and the lungs [85].

### 3.2. INF-λ Exhibit Antiviral Activity against Coronaviruses

Currently, there is little information on the role of the innate immune response in SARS-CoV-2 pathogenesis. Consequently, data on the induction of cytokine responses to beta-coronaviruses are of particular interest. It has been shown that MERS-CoV (strain HCoV-EMC) did not induce type I or type III IFN in human bronchial or lung tissue, while treatment of cells with IFN-α or IFN-β one hour after infection reduced viral replication [86]. It has also been shown that neither MERS-CoV nor SARS-CoV is able to induce expression of IFN-α/β in monocyte-derived macrophages. However, those viruses slightly increase the expression of pro-inflammatory IL-6, TNF-α, IFN-γ, and chemokines (IP-10, MCP-1, MIP-1α, RANTES, and IL-8). Interestingly, the expression of cytokines and chemokines was significantly higher with MERS-CoV infection than with SARS-CoV infection [87]. Another research group [88] evaluated the innate immune response of primary, type II epithelial cells infected with SARS-CoV. An increase in expression of IFN-β and IFN-λ, as well as pro-inflammatory cytokines and chemokines (IL-6, IL-8, IP-10, CXCL-11, RANTES), was noted one day after infection. Although pneumocytes and alveolar macrophages are considered to be the main targets for SARS-CoV-2, viral replication is not associated with the induction of type I or type III IFN in these cells. Increased expression of chemokines (MCP-1, CXCL-1, CXCL-5, IP-10) and IL-6 in ex vivo lung tissues has been reported [89]. Thus, SARS-CoV-2 induces a narrow group of pro-inflammatory cytokines and chemokines, and the absence of type I or III IFN expression may be a reason behind insufficient innate immune responses and high pathogenicity [90]. For the aforementioned reasons, the use of exogenous type I and III IFN can be considered a promising therapeutic approach for SARS-CoV-2 [90].

Type I and type III IFN have been shown to exhibit antiviral activity against SARS-CoV-2. Pretreatment of primary human airway epithelial cells with PEGylated IFN-λ1 decreased SARS-CoV-2 titers [70]. Using two mammalian epithelial cell lines (human Calu-3 and simian Vero E6), both IFN-α and IFN-λ have demonstrated dose-dependent inhibition of SARS-CoV-2. In contrast, an inhibitor of IFN-triggered JAK/STAT signaling (ruxolitinib) boosted SARS-CoV-2 replication in the IFN-competent Calu-3 cells [91]. The SARS-CoV-2 virus is also capable of productively infecting human intestinal epithelial cells. It was shown, in both T84 and Caco-2 cells, that treatment with IFN-β or IFN-λ one hour after SARS-CoV-2 infection resulted in inhibition of viral genome replication and non-release of de novo infectious viral particles. Moreover, *IFNLR1* knockout led to significantly increased SARS-CoV-2 replication and an increase in the number of viral particles by about three orders of magnitude. Similar results were obtained with an *IFNAR1–IFNLR1* double knockout. Moreover, a pan-JAK inhibitor (pyridone-6) caused augmented SARS-CoV-2 replication and an increase in the number of infected cells. This highlights the necessity for JAK/STAT1-mediated signaling pathways in the protection of human intestinal epithelial cells from SARS-CoV-2 infection [92]. In vivo, it has been shown that administration of PEGylated IFN-λ1 prevented Balb/c mice from losing weight and led to reduced SARS-CoV-2 lung titers on the second day post-infection; this was true in both a prophylactic scheme (treatment before infection) and a therapeutic scheme (treatment following infection) [70]. Therefore, there is currently some evidence that IFN-λ feature antiviral activity against SARS-CoV-2 in vitro and in vivo.

### 3.3. Antiviral Activity against Other Respiratory Viruses

Lambda IFN are well known for their antiviral activity against respiratory viruses. It has been shown in vivo that a double knockout of the IFNΑR1 and *IFNLR1* genes leads to increased susceptibility to influenza A virus (IAV), influenza B virus (IBV), respiratory syncytial virus, metapneumovirus, and SARS-CoV, with disturbance of IFN-λ-mediated signaling playing a key role [85]. A549 cells have been shown to express all four IFN-λ types in response to metapneumovirus infection, and IFN-λ1-3 in response to respiratory syncytial virus infection [5]. Preincubation of respiratory epithelial cells with IFN-λ1 reduced viral titer. A therapeutic scheme (treatment following respiratory syncytial virus infection), however, did not show any antiviral effects. This suggests that the virus has protective mechanisms that inhibit IFN-λ1 activity. Non-structural proteins NS1 and NS2 have been shown to be capable of suppressing JAK/STAT signaling cascades. This is reflected in decreased pSTAT2 (but not pSTAT1) levels, with consequent inhibition of MxA/B production [93]. Meanwhile, IFN-λ has shown encouraging results in animal studies: treatment of mice with IFN-λ2 and IFN-λ3 led to decreased viral titers in the lungs, attenuation of pulmonary inflammation, inhibition of pro-inflammatory cytokine and chemokine production, and higher survival of infected animals [5].

Rhinovirus is one of the main causes of asthma complications. Antiviral activity of IFN-λ1 is manifested in relation to both acute and persistent rhinovirus infection [94]. The virus is able to induce the expression of lambda IFN. However, the levels of these IFN in infected asthma patients are significantly reduced compared with healthy volunteers. Dysfunction of lambda IFN production inversely correlates with viral replication levels and inflammation; it is also associated with impaired lung function. Such data cumulatively indicate a protective effect of lambda IFN [41]. In a murine atopic asthma model, treatment with IFN-λ2 had several effects: reduced eosinophil and neutrophil numbers in the bronchoalveolar fluid (BALF), decreased leukocyte infiltration into the lungs, decreased mucus secretion, and diminished Th2- and Th17-mediated immune response in the lymph nodes despite an activated IFN-γ-inducible response [42]. Moreover, knockout of the *IFNLR1* gene negated these protective effects and decreased IFN-γ production in the lymph nodes [42]. Therefore, IFN-λ2 exhibits immuno-modulatory activity: inhibiting Th2- and Th17-dependent differentiation of T cells, yet enhancing Th1-differentiation and promoting IFN-γ-mediated response. The cellular mechanism of the regulation is based on IFN-λ2′s influence on CD11c^+^ dendritic cells and bone-marrow-derived dendritic cells. Upon stimulation with IFN-λ2, dendritic cells express the T-bet transcription factor associated with Th1-polarization. Moreover, IFN-λ2 induces the production of IL-12p70, which also causes Th1-differentiation; IFN-λ2 further reduces the expression of OX40L, a costimulator of Th2-differentiation. Knockout of the *IFNG* gene negates IFN-λ2′s protective effects, which can also be blocked by IL-12-specific antibodies, as inhibition of IL-12 diminishes the production of IFN-γ in T-cells. This highlights the importance of both cytokines in the realization of an IFN-λ2-mediated shift in Th1/Th2 balance [42].

It may be concluded from the aforementioned that lambda IFN exhibit distinct immuno-modulatory activity. In PBMC, lambda IFN provoke activation of Th1-mediated response and suppression of Th2-mediated response. In a murine asthma model, it was shown that treatment with IFN-λ3 increased the expression of Th1 cytokines and decreased Th2 cytokines. This matches with data that SNP in the *IFNL3* gene are associated with an increase in eosinophil numbers and Th2 cytokine levels [29]. IFN-λ1 also displays immuno-modulation. It mainly inhibits the production of IL-13; it also, to some extent, reduces the production of IL-4 and IL-5 in PBMC obtained from healthy donors [95]. Moreover, it was shown that IFN-λ1 suppressed IL-4, IL-5, and IL-13 (but not IL-10 or IFN-γ), decreased eosinophil numbers in BALF, reduced mucus production, and reduced serum IgE titer. Additionally, IFN-λ1 administration activated CD4^+^/CD25^+^/Foxp3^+^ regulatory T cells that suppress the Th2- and Th17-dependent responses. Thus, IFN-λ1 promotes a shift in Th1/Th2 balance [96]. Furthermore, IFN-λ1 increased the production of IL-12p40 in a population of macrophages differentiated from monocytes; IFN-λ1 activated macrophages were more susceptible to IFN-γ stimulation and produced IL-12p40 and TNF-α [29]. It has been shown that CD4^+^ T cells are susceptible to IFN-λ1, and expression of the GATA3 and Th2 cytokines (IL-4 and IL-13) was suppressed by IFN-λ1. However, IFN-λ1 did not affect cell proliferation or the Th1 response [97]. Consequently, IFN-λ1 affects the development of naive T cells and shifts the Th1/Th2 balance towards Th1.

## 4. The Role of IFN-λ Specifically during Influenza Virus Infection

### 4.1. Influenza Virus Infection and Respiratory Airway Epithelium

Infiltration of influenza virus into a host leads to infection of type II epithelial cells lining the respiratory tract, and subsequent development of inflammatory processes. In mild cases, infection is limited to the upper respiratory tract (URT). In cases featuring severe pathologies, infection can penetrate into lung tissues [39,98,99,100]. The alveolar epithelium is responsible for gas exchange, and it is the main target of IAV and influenza-induced pneumonia [101]. Respiratory tract epithelia are known to be covered with mucus. Therefore, virions must pass through the mucus layer to reach the target cells [102]. Epithelial cell surfaces contain sialic acid receptors (SAR), which are sialyl oligosaccharides. The influenza virus hemagglutinin (HA) protein is able to bind to SAR, thereby permitting the virion to enter the epithelial cell through endocytosis. Accordingly, viral infiltration depends on SAR densities on respiratory epithelial cells and on interaction affinities with the various HA types. The interaction of HA with SAR is specific, and human influenza virus strains mainly interact with α-2,6-SAR, which are most prevalent in the URT. However, α-2,3-SAR sialyl oligosaccharides bind mainly to avian influenza virus strains, and they are most common in the lower respiratory tract. Thus, URT epithelial cells are the main targets of human influenza virus strains [103]. Through the use of traceable IAV (a modified A/Puerto Rico/8/34 (PR/08) strain with a green fluorescent protein within the viral genome NS segment), it was shown that IAV infection begins in the trachea and the main bronchi, and spreads to bronchiolar regions and, possibly, alveoli. Spread of influenza virus in the lungs depends on a wide variety of factors, including: virus origin (human, avian, etc.); HA specificity; accessibility of target cells and SAR; and even body temperature [104]. In early-stage infection with Balb/c mice, accumulation of viral NS protein has been observed in non-immune CD45^−^ cells of the respiratory tract. Expression of IAV proteins was detected in hematopoietic cells only a day after infection [104]. Consequently, epithelial CD45^−^cells of the trachea and major bronchi are the primary targets of IAV in mice.

Type II alveolar cells synthesize and secrete pulmonary surfactant, chemokines, and cytokines; they are involved in the innate immune response of the lungs [39,101]. As such, infection with IAV (strain PR/08) induces secretion of IL-6, IL-8, MIP-1, RANTES, MCP-1, IL-10, and IFN-β, but neither IFN-α nor IFN-γ. IL-8 and MCP-1 are known to be the main stimulants for neutrophil and monocyte migration, respectively, in acute lung inflammation. However, it should be noted that IFN-λ1 is the main IFN induced by IAV infection in type II alveolar epithelial cells [39]. The pro-inflammatory cytokines and chemokines, together with PRR ligands, stimulate the production and activation of immune cells [103]. The host’s immune response varies greatly depending on the influenza virus strain. For instance, it was shown that infection with IAV H5N1 led to more pronounced induction of pro-inflammatory cytokines and chemokines (IP-10, IFN-β, IL-6, RANTES) in human primary epithelial cells, compared with infection with H1N1 [105]. The induction of pro-inflammatory cytokines and chemokines is correlated with viral strain pathogenicity. More pathogenic strains are able to induce higher levels of cytokines and chemokines [103].

IAV infection can activate pattern-recognition receptors (PRR) such as viral nucleic acid sensors. In one example, the “nucleic acid” is not coming only from the invading virus, but also from endogenous retroviruses (ERV) that are no longer repressed by the TRIM28 restriction element, because of an IAV-induced mechanism to suppress TRIM28 [106]. IAV causes the small ubiquitin-like modifier (SUMO) to modify TRIM28, thereby inactivating its restriction abilities and unleashing ERV RNA into the cell. Such RNA would ordinarily induce innate responses like IFN production were it not for the viral nucleic-acid binding protein NS1 that is able to soak up the excess RNA.

With influenza virus infection, a significant similarity in the functioning of type I and type III IFN has been noted [107,108]. While dendritic cells and alveolar macrophages mainly produce type I IFN, respiratory epithelial cells are the main producers of type III IFN. Induction of both types in virus-infected epithelial cells occurs in response to the same antigenic determinants and requires the activation of the same signaling pathways. Moreover, they induce almost identical sets of ISG [107,108]. IFNαR is expressed ubiquitously, while IFNλR1 subunit expression is observed mostly in epithelial cells of the respiratory and gastrointestinal tracts. During influenza virus infection, IFN-λ provides respiratory epithelial cells with an antiviral response and also limits direct activation of immune cells associated with the development of uncontrolled inflammatory processes in the lungs [6,39,108].

### 4.2. Knockout Mouse Models

To determine the involvement of a gene in the control of a viral infection, it is convenient to use models featuring a knocked out gene or group of genes. Thus, more than a decade ago, the involvement of IFN-λ-dependent signaling pathways in the suppression of influenza virus infection was shown. *IFNLR1* gene knockout caused an increase in lung viral titers, but did not affect the survival curves of infected mice. Combined knockout of the *IFNAR* and *IFNλR1* genes led to a significant increase in mortality, hypersensitivity (even to non-pathogenic strains lacking NS1), significant increases in viral titers, and blockage of Mx1 production. In addition, intranasal administration of recombinant IFN-λ2/3 protected *IFNAR* knockout animals from lethal doses of IAV (subtype H7N7) [109]. It has been also shown that mice with an *IFNL2–IFNL3* double knockout are characterized by impaired control of viral replication in the lungs and exhibit a phenotype similar to that observed with *IFNLR1* gene knockout [110]. The importance of IFN-λ-dependent signaling in the suppression of influenza virus infection has been shown in a number of other works [72,85,107,111] however IFN-λ was considered to be an auxiliary antiviral protective mechanism, acting in parallel with IFN-α/β [107]. Later, it was shown that IFN-λ, unlike IFN-α/β, does not cause the development of an uncontrolled pro-inflammatory response, or “cytokine storm” [108], which is one of the main causes of influenza virus infection pathology [103,112].

It has recently been demonstrated that the greatest differences between IFNλR1-defective mice and wild-type mice are observed during infection with low doses of influenza virus [110,111,113]. In particular, it was shown that *IFNLR1* knockout was associated with a significant viral infiltration from the URT into the lungs and an increase in viral titer (lung, URT) upon infection with IAV SC35M (H7N7) and Udorn (H3N2). It should be noted that the development of an influenza virus infection depends on the dose of the injected viral inoculum. A high dose can lead to viral infiltration into the lungs (which is associated with a greater lethality of the model) and to the development of a “cytokine storm” hiding the protective effects of IFN-λ [113]. Correspondingly, a four-fold smaller inoculum dose permits selective infection of URT cells, thereby mimicking a natural course of the infection [113]. Mice with *IFNAR1–IFNLR1* double knockout, as with *IFNLR1* knockout alone, were characterized by increased viral loads at 3–5 days after infection, increased levels of pro-inflammatory immune cells in BALF (mainly neutrophils and macrophages), increased pro-inflammatory cytokine and chemokine levels (IFN-α, IL-6, CCL2, CCL3, CXCL1/keratinocyte chemoattractant (KC)), and lung tissue damage. At the same time, animals with single *IFNΑR1* knockout did not differ from wild-type mice in resistance to viral challenge, and they were characterized by reduced production of pro-inflammatory, first-wave cytokines and chemokines. These data prove the importance of IFN-λ in the suppression of sub-lethal influenza virus infection [111]. In turn, with a lethal viral dose, knockout of *IFNΑR1* becomes a key factor, while IFNλR1-deficient mice did not differ from the wild-type in viral load from the 3rd day after infection. With a lethal viral challenge, *IFNLR1* knockout was associated with neutrophil infiltration, increased levels of IFN-α, CCL3, and CCL4, and impaired pulmonary function. These effects may be associated with either impaired control of viral replication or rather with IFN-λ immuno-modulatory activities [111].

Therefore, it can be concluded that IFN-λ and IFN-α/β are two complementary, non-redundant types of IFN for controlling influenza replication. The first type is implemented in the early stages of infection and is effective against sub-lethal doses of the virus, while IFN-α/β are activated at later stages and are effective against high doses of the virus; their action, however, is associated with the development of a systemic inflammatory process [111,113]. The mouse *IFNAR–IFNLR1* double knockout model resembles the *STAT1–STAT2* double knockout model, which emphasizes the unique role of type I and III IFN in inducing ISG expression and generating the innate antiviral response [72]. At the same time, IFN-λ levels in BALF and pDC of IAV (A/WSN/33 (H1N1)) infected mice exceed IFN-α/β by more than tenfold [72]. Such a significant expression of IFN-λ allows one to speculate that these IFN are the key components of the innate immune response against influenza virus infection.

### 4.3. Induction of IFN-λ in Influenza Virus Infection

The main mechanism of IFN-λ induction is influenza-induced PRR activation [107,114]. Notably, some research indicates that IFN-λ production is a response to the infiltration of live viruses specifically. Thermal inactivation of IAV (65 °C for 30 min.) decreased elicited IFN-λ production to the level of non-infected controls [73,107]. Such inactivation denatures HA and prevents host cell attachment [73], while inactivating viral polymerase [107]. Thus, IFN-λ expression is associated with infiltration of live viruses into host cells and replication of the viral genome [5,73,107]. It is known that IAV infection induces the expression of all IFN-λ subtypes [114]. It has recently been shown that IFN-λ1 is produced in most IAV-infected cells (strain PR08), while expression of the remaining type I and III IFN is limited to a relatively small group of cells [114]. Perhaps, a paracrine IFN-λ1 induction mechanism occurs that is based on intercellular communication [114]. At the same time, *IFNAR1* gene knockout is associated with a decrease in basal expression of IFN-λ2 and IFN-λ3 [62,113], which can be explained by the absence of the IFN-β-dependent loop for positive regulation of IFN-λ expression. *IFNLR1* knockout, in contrast, does not significantly affect IFN-α/β levels [62]. Therefore, it can be assumed that there is a positive—autocrine or paracrine—loop of IFN-λ induction that is mediated by IFN-β.

Influenza infiltration into host cells is related to activation of the RIG-I/MAVS signaling pathways. Infection of A549 cells or alveolar epithelial cells with IAV (strain PR08) promotes the dose-dependent expression of RIG-I and TLR-3, but not TLR-7 or NOD2. Activation of RIG-I and TLR-3 is associated with the expression of IFN-λ, as well as IFN-β and IP-10 [115]. *DDX58–TLR3* double knockdown led to: a decrease in IRF-3 phosphorylation; almost complete blockage of IFN-λ1 and IFN-β production; and no significant changes in IFN-λ2 and IFN-λ3 expression [115]. Nevertheless, IFN-λ expression is known to be associated with RIG-I expression during influenza virus infection [40,73,116]. Using CIAP (calf intestine alkaline phosphatase), which is used to remove the triphosphate group from the 5′-end of viral RNA, a decrease in RIG-I-mediated signaling pathway activation was detected, leading to inhibition of IFN-λ expression [73]. Moreover, *DDX58* silencing also markedly suppressed IFN-λ production in A549 cells [73]. Meanwhile, it was found that knockout of *TLR7*, its molecular adapter *MYD88*, or *TICAM1*, did not affect the production of IFN-λ2/3 in murine tracheal epithelial cells (MTEC) cells [107]. In addition, MAVS-deficient cells were not able to produce sufficient IFN-λ2 and IFN-λ3 in response to influenza virus infection [107]. Overall, these data emphasize the role of the RIG-I/MAVS signaling pathway in IFN-λ expression [107].

The expression of IFN-λ is mediated by the activation of several transcription factors. Knockout of *IRF3* did not block the ability of MTEC to produce either IFN-λ2/3 or IFN-β [107]. It was also shown that *IRF3–NFκB* double knockdown inhibited poly(I:C)-mediated IFN-λ induction [117]. With *IRF7* knockout, a significant decrease in type I and III IFN expression has been shown [107]. In response to IAV infection, however, IFN-λ expression was completely blocked only with a *IRF3–IRF7* double knockout. MTEC cells with *IFNAR1–IFNLR1* double knockout were able to produce both IFN-λ2/3 and IFN-β in amounts comparable to the wild-type. However, ISG expression *(OASL-1, RSAD2, IFI3*) was completely suppressed [107]. Therefore, it is reasonable to conclude that the synergistic action of several transcription factors (IRF-3, IRF-7, NFκB) is required for optimal IFN-λ induction in response to influenza virus infection [107].

Induction of IFN-λ can also be caused by the close cooperation of respiratory epithelial cells with bacterial microflora of the URT. Viral infections can induce an ideal environment for a bacterial superinfection through different mechanisms such as the destruction of the epithelial barrier, the over-expression of the receptors involved in the bacterial adhesion to the cells, and the alteration of the host immune response [118]. On the other hand, certain commensal bacterial exhibit antiviral effects. *Staphylococcus epidermidis* is the most common commensal bacteria of the healthy nasal mucosa [119]. Under stimulation with *Staphylococcus epidermidis*, normal human nasal epithelial cells are capable of TRL-2-independent IFN-λ production and expression of antiviral ISG (*CXCL10*, *IFIT1*, *MX1*, *OAS1*), which facilitate the suppression of IAV replication. However, IFN-α,-β, or -γ production is not observed with such stimulation. Silencing of *IFNLR1* with short hairpin RNA attenuates the antiviral effect of commensal bacteria, which indicates the specificity of IFN-λ-dependent signaling pathways. Moreover, *Staphylococcus epidermidis* has been shown to prevent the spread of IAV to the lungs by stimulating IFN-λ production and suppressing viral replication in the nasal mucosa. Intranasal administration of commensal bacteria promotes the survival of mice. It seems to be important that administration of *Staphylococcus epidermidis* in that research was not associated with bacterial infiltration into the lungs, and IFN-λ levels in BALF were not increased. Thus, *Staphylococcus epidermidis* is part of the first line of defense against influenza virus infection, and its activity modulates IFN-λ production in the nasal mucosa [119].

### 4.4. The Antiviral Effect of IFN-λ in Influenza Virus Infection

It has been shown that type I IFN and IFN-λ can independently induce antiviral ISG [21,61,107,108,111,120]. In the absence of both mechanisms of antiviral protection, a loss of ability to induce ISG is observed, which indicates a lack of additional mechanisms for the activation of these genes [107]. The antiviral effect of IFN-λ against various influenza virus subtypes has been shown. In type II alveolar epithelial cells infected with IAV (PR/08), IFN-λ1 induced antiviral ISG (*MX1*, *OAS*, *ISG56*) in a dose-dependent manner. Moreover, IFN-λ1 reduced viral release and suppressed influenza-induced secretion of chemokines (MIP-1β, RANTES, MCP-1 and IL-8). IFN-λ1 also attenuates IFN-β expression [39]. One day after infection of MTEC with IAV (PR/08), expression of genes associated with activation of IRF and IFN signaling was noted. A significant increase in IFN-λ2/3 and a slight increase in type I IFN (IFN-β, IFN-α4, IFN-α5) levels was shown [107].

Antiviral IFN-λ activity was more pronounced with an avian IAV strain (A/chicken/Germany/27) than with the human PR/08 strain [121]. In A549 cells, IFN-λ1,2,3 reduced avian IAV (H7N7) titer and showed the ability to induce IFN-β expression. Interestingly, IFN-λ1,2,3 decreased the expression of RIG-I; type I IFN, on the contrary, increased. Avian H7N7 did not affect the RIG-1 level [122]. In IBV-infected A549 cells, there was a significant increase in the expression of IFN-λ and canonical antiviral ISG (*MX1*, *OAS*, *IFITM1*), but not IFN-α/β. Peak IFN-λ expression occurred 8 h after infection. IFN-λ, in a dose-dependent manner, inhibited IBV replication [61].

Compared with IFN-α and IFN-ω, IFN-λ more effectively reduces viral load, which may be due to differences in ISG induction kinetics [121]. It was shown that the level of IFN-λ-induced STAT1 phosphorylation was significantly reduced in IAV-infected A549 cells, and already 15 h after infection, it was indistinguishable from those of intact cells. Therefore, IAV is able to inhibit JAK/STAT-dependent signaling pathways. One of the possible inhibitors is the SOCS-1 protein [21], whose peak expression is observed approximately 10 h after infection. SOCS-1 can be induced by a cytokine-independent mechanism [73]. It has also been shown that infection of human tracheobronchial epithelial cells with IAV (subtypes H5N1 and H3N2) is associated with a significant increase in the expression of SOCS-1 and SOCS-3. This may be one of the mechanisms influenza exploits to avoid the host innate immune response [123]. Additionally, an inverse correlation between the expression of IFN and viral NS1 is seen in A549 cells, which may serve as indirect evidence of IFN response inhibition through NS1 protein [114]. Interestingly, pre-treatment of respiratory epithelial cells with IL-17A led to a decrease in poly (I:C)-mediated induction of IFN-λ, both at the mRNA and protein levels. Additionally, IL-17A is able to inhibit IAV (H3N2) induced IFN-λ production. Investigation of IL-17A’s inhibitory effects showed that it has a prolonged action and reduces IFN-λ levels, even two days after administration into the medium. Moreover, the addition of IL-17A inhibited STAT1 phosphorylation for up to 24 hrs after treatment. Thus, suppression of JAK/STAT-dependent signaling pathways may be one of the mechanisms by which IL-17A’s inhibitory effects are realized. IL-17A activity is mediated through the SOCS-1 and SOCS-3 proteins [117]. These data are interesting examples of the relationship between IFN-λ and other cytokine immuno-modulators.

### 4.5. Immuno-Modulatory Effects

IFN-λ is known to possess immuno-modulatory activities during infection with respiratory viruses, mainly rhinovirus and respiratory syncytial virus [29,42,93]. There are nuances specific to the influenza virus. It has been shown that IFN-λ3 polymorphism rs8099917 may be associated with influenza vaccination effectiveness. The presence of minor alleles at this polymorphism is associated with a decrease in IFN-λ3 level, which influences the Th1/Th2 balance. In response to IAV stimulation, PBMC of patients with the minor alleles were characterized by decreased Th1-cytokines and increased Th2-cytokines; HLA-DR expression in B lymphocytes was also increased. Moreover, recombinant IFN-λ3 in PBMC led to: increased IFN-γ and IL-6; inhibited expression of IL-4, IL-5, and IL-13; and reduced IAV-inducible proliferation of B cells and IgG production [29]. However, other researchers have shown that B cells are capable of expressing IFNλR1 and, conversely, IFN-λ increases TLR-7-mediated activation of B cells, causing immunoglobulin production [124]. Moreover, it was shown that murine IFN-λ2 is able to activate virus-specific CD8^+^ T cells and induce thymic stromal lymphopoietin (TSLP), an adaptive immune response regulator, in respiratory epithelial M cells [125]. Interestingly, the induction of TSLP in M cells is specific to IFN-λ, but not type I IFN. TSLP activates antigen presentation by CD103^+^ migratory dendritic cells and biases the immune response towards IgG1 and IgA production. The use of IFN-λ2 as an adjuvant is able to enhance the protective effect of a vaccine against IAV (PR08 and A/Udorn/72) [125]. To date, some contradictions regarding IFN-λ’s effect on the humoral immune response against influenza remain unresolved [29,125]. A possible cause may be differences in the functioning of the adaptive immune response in humans and mice. Additional research is required to resolve these contradictions.

The relationship between IFN-λ and the development of a specific, CD8^+^ T cell response has been shown with heterosubtypic IAV challenge. Neutralizing antibodies produced by B cells are known to protect against only one IAV subtype, but are not effective against infection with other subtypes. In contrast, the CD8^+^ T cell response is efficient, regardless of IAV subtype. Knockout of the *IFNLR1* gene led to an increased susceptibility to H1N1 (strain PR/08) in mice that had previously been infected with the IAV-X31 reassortant strain (with HA and NA from A/HK/1/68 (H3N2)). It was noted: a decreased production of IFN-γ and TNF-α on day 9 after infection with the PR/08 strain, and a reduction in the number of IAV-specific CD4^+^ and CD8^+^ T cells in the lung lymph nodes of *IFNLR1*-defective mice, 35 days post-challenge. Defects in the CD8^+^ T cell response are associated with impaired cellular memory and antigen priming. It has been shown that IFN-λ-dependent signaling is required in pulmonary dendritic cells (CD103^+^ dendritic cells and CD8α^+^ dendritic cell sub-populations) for maturation, migration to lymph nodes, upregulation of costimulant molecules, and antigen presentation in acute influenza virus infection. On the 4th day after infection, when an accumulation of pulmonary dendritic cells in the lymph nodes was observed (necessary for the CD8^+^ T-cell response), it was noted that *IFNLR1* knockout leads to upregulation of the LOC101055769, *RGS9BP*, *TMEM246*, *WRD31*, and *IL10* genes. Therefore, in dendritic cells, IFN-λ is required to inhibit IL-10 production, the immuno-modulatory effect of which is associated with impaired CD8^+^ T cell priming [126]. In addition, IFN-λ enhanced IDO1 activity, and *IFNL* knockdown decreased IDO1 expression during influenza virus infection [127].

It has also been shown that overexpression of IFN-λ3 led to an increase in the number of natural killer cells in the spleen, liver, and lungs. IFN-λ3 promoted the proliferation and maturation of these immune cells and contributed to the suppression of influenza (PR/08) infection in mice. It was shown that the IFN-λ3′s effect on natural killer cells is mediated by activation of macrophages which, in turn, influence natural killer cells [128]. In neutrophils, high levels of the *IFNλR1* and IL10R2 subunits are observed, which makes these cells, along with epithelial cells, the main targets of IFN-λ. Neutrophil activation through IFN-λ is neither associated with significant infiltration of these immune cells into the lungs nor a significant increase in the level of pro-inflammatory cytokines. Gene expression data (RNA-seq) revealed that IFN-λ and IFN-α affect neutrophils, including induced expression of an extensive set of ISG (*ISG15*, *ISG20*, *OAS1*, *OASL1*-2, *IFI47*, *IFIT1*-3, *RSAD2*) and PRR (*DDX58*, *IFIH1*, *TLR3*, *TLR7*). IFN-α, however, was additionally characterized by stimulation of pro-inflammatory cytokine and chemoattractant production (*TNF*, *IL1b*, *IL6*, *CCL2*, *CCL4*, *CCL5*, *CXCL9*-11). Therefore, it is possible that one of the functions of respiratory-epithelium-produced IFN-λ is the protection of neutrophils that infiltrate the lung during influenza virus infection [111]. Another study noted no significant neutrophil infiltration into the lungs [108]. It is also important to note that, in PBMC, IFN-λ does not significantly increase the expression of certain ISG (*IRF7*, *RSAD2*, *OAS1*), nor does it lead to the secretion of high amounts of certain pro-inflammatory cytokines or chemokines (IL-6, MCP-1, IP-10); this contributes to the localization of the inflammatory process [108].

### 4.6. Use of Recombinant IFN-λ: A Two-Faced Janus

A significant increase in IFN-λ expression has been shown in response to IAV infection in vivo [62,73]. Murine models are extensively used to investigate the specific effects of type I and III IFN. In vivo studies have shown that IFN-α/β treatment yielded contradictory results [129]. This is associated with the pro-inflammatory effects of type I IFN: recruitment of innate immune cells to the site of infection (mainly pDC and monocytes); increases in pro-inflammatory cytokines and chemokines in BALF (IL-6, IP-10, MCP-1, MIP-1α); and increases in the frequency of respiratory epithelial cell apoptosis. However, it has been shown that treatment with either IFN-α or IFN-λ is able to completely suppress influenza virus infection in the murine lung [108]. As such, the intranasal administration of recombinant IFN-λ2 has a protective effect against IAV (PR/08). It is notable that there was no increase in pro-inflammatory cytokine levels, and the action of IFN-λ2 protected pulmonary epithelium from virus-induced apoptosis. As part of an analysis of genes induced by IFN-α or IFN-λ, it was shown that a cluster of genes exists, whose expression is specifically induced by IFN-α and which is associated with immune cell recruitment, hypercytokinemia, and hyperchemokinemia [108,111]. This contributes to the development of a “cytokine storm” and inflammatory processes in lung tissue [103,112]. Therefore, IFN-α/β is a powerful immuno-modulatory mechanism [107], and the addition of exogenous IFN-α/β can lead to detrimental outcomes, whereas IFN-λ lacks at least some of the adverse effects [108,111].

It has been shown that viral load is the key factor determining the features of the innate immune response [111]. Administration of PEGylated IFN-λ2 in a sub-lethal model of influenza virus infection suppressed viral replication and decreased the number of pro-inflammatory immune cells in BALF. It also reduced a number of other factors: pro-inflammatory cytokines and chemokines (TNF, IFN-γ, CCL3, CCL4, CXCL9, IP-10); as well as type I IFN. It has also been shown that both IFN-α and IFN-λ reduced IAV (subtype H3N2) titer in the lung [113]. However, IFN-λ is able to inhibit viral replication in the URT to a much greater extent than IFN-α, and IFN-λ reduces the risk of transmission of the virus from one animal to another. Moreover, the antiviral effects of IFN-α against IAV in the URT were not apparent three days after intranasal administration. The effects of the IFN-λ, however, were still present, and MxA expression reached its peak only a day after administration [113], which correlates with IFN-λ3 expression kinetics [29]. These data cumulatively indicate the unique value of IFN-λ in antiviral protection precisely in UTR tissues [113].

In addition to inhibiting influenza infection, IFN-λ may reduce the risk of some complications. The presence of allergic rhinitis may be associated with increased susceptibility to influenza infection. One of the explanations is the disturbance of IFN-λ-dependent signaling pathways. Thus, in patients with allergic rhinitis, the IFN-λ expression in nasal mucosal cells is attenuated, and recombinant IFN-λ reduces viral load in allergic rhinitis nasal epithelial cells. Using an allergic rhinitis model, IFN-λ has been shown to reduce viral load in the nasal mucosa of IAV-infected mice [130].

The aforementioned supports the use of IFN-λ in the treatment of influenza infection. However, questions about the possible risks of such an approach have appeared in recent years. The risks are related to the anti-proliferative properties of IFN-λ. The proliferation of type II respiratory epithelial cells is observed 6–8 days after infection, which correlates with the time frame of influenza elimination and weight gain. It has been shown that in this time period IFN-λ, but not IFN-α/β, is present in BALF. IFN-λ inhibits the proliferation and differentiation of pulmonary epithelium. Moreover, single knockout of the *IFNAR1* or *IFNLR1* genes improved cell proliferation compared to wild type [62], while not significantly disrupting suppression of viral replication by innate immunity due to the parallel effects of IFN-α/β and IFN-λ [107,109]. IFN-β and IFN-λ have the highest antiproliferative effects, which is associated with: impaired cell cycle promotion, and apoptosis. These effects, however, were observed only in actively dividing cell cultures [62]. The antiproliferative effects of IFN-β and IFN-λ are associated with upregulation of the p53 tumor suppressor protein, with IFN-λ inducing a more significant increase in p53. The anti-proliferative effect of IFN-λ can lead to increased susceptibility to bacterial pathogens, such as Streptococcus pneumonia [62].

It is worth noting that the period of maximum susceptibility to secondary bacterial lung infections occurs on the 6–7th day after influenza infection. *IFNLR1* knockout mice were characterized by: increased expression of IL-22 and Ngal; lower URT bacterial load; and less susceptibility to bacterial pneumonia [131]. Some research presents data that IFN-λ administration leads to complications in cases of mixed infection [132]. In that study, IFN-λ administration was carried out on the fifth day after infection with IAV (strain PR/08); one day after administration, mice were infected with *Staphylococcus aureus* or *Streptococcus pneumoniae*. Despite an increase in URT bacterial load, it was found that IFN-λ did not inhibit the expression of IL-17, IL-22, Ngal, or pulmonary antimicrobial peptides. Perhaps the deleterious effect of IFN-λ in mixed infections is based on a decrease in neutrophil mobility and, consequently, a decrease in the number of neutrophils recruited to lung tissue. Attenuated levels of neutrophil chemo-attractants (CXCL1/keratinocyte chemoattractant (KC), granulocyte colony-stimulating factor (G-CSF), IL-1α) in lung tissue homogenates may be a convincing indication of this assumption. It has also been shown that IFN-λ inhibits bacterial phagocytosis by pulmonary neutrophils. Interestingly, an increase in bacterial load and a decrease in the number of neutrophils in BALF was detected only with mixed infection, but not with *Staphylococcus aureus* infection alone. It can be assumed that IFN-λ, acting in combination with IAV-induced cytokines, can inhibit the activity of neutrophils [132]. Therefore, although a decrease in neutrophil recruitment to the lungs has a protective effect during influenza infection, it should be taken into account that neutrophils play a critical role in the suppression of secondary bacterial infections caused by *Staphylococcus aureus* or *Streptococcus pneumoniae* [133].

## 5. Conclusions

Lambda IFN are important mediators of the innate immune response and, along with type I IFN, form the primary line of defense against viral infections [108]. In contrast to both IFNAR subunits, expression of IFNλR1 is limited to a relatively narrow group of cells primarily mucosal epithelial cells [30]. This ensures the development of an IFN-λ-dependent antiviral response in respiratory epithelium which is not accompanied by direct activation of immune cells [108]. In contrast, IFN-α/β action is associated with the development of uncontrolled inflammatory processes in the lungs and occasionally, “cytokine storm” [112]. Therefore, IFN-λ can be considered a promising therapeutic agent against respiratory infections [26,30]. For example, some researchers tested IFN-λ as a therapeutic agent against SARS-CoV-2 [70,90]. On the other hand, IFN-λ has an ambiguous role in the suppression of influenza infection: it has a protective effect in the early stages of the illness [108,111,113], but has a negative influence on epithelial tissue recovery and increases the risk of bacterial secondary infections [62,132]. Therefore, IFN-λ can serve as a therapeutic agent only in the early stages of the influenza-induced inflammatory process, and regulation of IFN-λ expression may be considered a key mechanism for influenza therapy.

## Figures and Tables

**Table 1:** Viruses affected by IFN-lambda (Type III interferons).

*Virus Family* Common Names	Virus Genome	Infected Cells Expressing IFN-λ and IFN λR	Effects of IFN-λ
*Myxoviridae* Influenza A Influenza B	− strand ssRNA	Respiratory epithelia, keratinocytes, mDC and pDC, hepatocytes and primary neuronal cells; NOT macrophage	IFN-λ decreases influenza virus replication in a dose-dependent manner in respiratory and gastrointestinal epithelial cells by up-regulating ISG (*MX1*, *OAS*, *IFITM1*) [61]. IFN-λ is more anti-proliferative and anti-inflammatory than IFN α/β [62]. Anti-proliferative effects due to up-regulation of p53 can increase susceptibility to bacterial pathogens [62].
*Paramyxoviridae* Resp. syncitial virus (RSV); Metapneumovirus Measles virus	− strand ssRNA	Respiratory epithelia	Mice treated with IFN-λ2 and -λ3 had decreased viral titers, less pulmonary inflammation, and higher survival rates [5]. Metapneumovirus replication is attenuated in DC through MDA-5-mediated IFN response [44]. IFN-λ restricts measles replication in lung epithelial cells [63].
*Arenaviridae* Lymphocytic choriomeningitis virus (LCMV)	− strand ssRNA	Respiratory epithelia, DC	IFN-λ2 and -λ3 elicit an antiviral effect against LCMV in lung cell culture [64].
*Flaviviridae* Hepatitis C Dengue virus	+ strand ssRNA	Primary hepatocytes DC and Lung epithelial cells	Successful Heptitis C treatment is associated with human genetic SNPs in IFN-λ3 promoter [65] and in IFN-λ4 [28]. IFN-λ1 reduced DC migration by reducing CCR-7 expr [66]. IFN-λ1 and -λ2 increase antiviral ISG (OAS and Mx1) and thus decrease virus loads [67].
*Caliciviridae* Norovirus (NoV)	+ strand ssRNA	Intestinal epithelia	IFN-λ clears persistent NoV, affects gut microbiota, and prevents transmission of acute NoV [68].
*Picornaviridae* Rhinovirus	+ strand ssRNA	Respiratory epithelia, DC	IFN-λ decreases rhinovirus replication and the asthmatic effects of rhinovirus. In a murine model for asthma, treatment with IFN-λ2 reduces Th2, eosinophils and neutrophils in bronchial fluid [30,41,42].
*Picornaviridae* Coxsackie virus	+ strand ssRNA	Primary human hepatocytes	Coxsackie titers were 10–100X lower in IFN-λ-treated cells [69].
*Coronaviridae* MERS-CoV SARS-CoV-1 and -2	+ strand ssRNA	Respiratory epithelia	The coronaviruses induce little type I or type III IFN, but treatment with PEGylated IFN-λ1 decreased SARS-CoV-2 titers [70].
*Herpesviridae* Cytomegalo- virus (CMV) Herpes (HSV-1 HSV-2)	dsDNA	CMV infects human foreskin fibroblasts. HSV infects buccal or genital mucosa	IFN-λ3 lowers infection with CMV [49]. IFN-λ lowers infection with HSV-1 [48].
*Hepadnaviridae* Hepatitis B (HBV)	+ strand ssDNA	HBV infects primary hepatocytes.	HBV infection up-regulates expression of IFN-λ [26]. IFN-λ1 significantly reduced viral load during infection with HBV [71].

## Abbreviations

BALF	bronchoalveolar fluid
HA	hemagglutinin
HBV	hepatitis B virus
HCV	hepatitis C virus
HSV	herpes simplex virus
IAV	influenza A virus
IBV	influenza B virus
IFN	interferon(s)
IFNαR	type I (α/β) interferon receptor
IFNαR1	interferon-α/β receptor subunit 1
ISG	interferon stimulated gene(s)
mDC	myeloid dendritic cell(s)
MTEC	murine tracheal epithelial cell(s)
pDC	plasmacytoid dendritic cell(s)
RLR	RIG-I-like receptor(s)
PRR	pattern recognition receptor(s)
SAR	sialic acid receptor(s)
SARS-CoV	severe acute respiratory syndrome coronavirus
TLR	Toll-like receptor(s)
URT	upper respiratory tract
